# Effect of questionnaire structure on recall of drug utilization in a population of university students

**DOI:** 10.1186/1471-2288-9-45

**Published:** 2009-06-29

**Authors:** Helena Gama, Sofia Correia, Nuno Lunet

**Affiliations:** 1 Department of Hygiene and Epidemiology, University of Porto Medical School, Porto, Portugal; 2 Institute of Public Health – University of Porto (ISPUP), Porto, Portugal

## Abstract

**Background:**

Self-reported data are a common source of information about drug exposure. Modes of data collection differ considerably and the questionnaire's structure may affect prevalence estimates. We compared the recall of medication use evaluated by means of two questionnaires differing in structure and length.

**Methods:**

Drug utilization was assessed by two alternative versions of a questionnaire (A – 4 pages, including specific questions for 12 indications/pharmacological groups and one question for "other medicines"; B – 1 page, including 1 open-ended question to cover overall drug consumption). Each of 32 classes in a private University in Maputo, Mozambique, was randomly assigned questionnaire A (233 participants) or B (276 participants). Logistic regression (allowing for clustering by classroom) was used to compare the two groups in terms of socio-demographic characteristics and medication used during the previous month.

**Results:**

Overall, 67.4% of the subjects had used at least one drug during the previous month. The following prevalences were greater among participants completing questionnaire A: use of drugs from two or more pharmacological groups (60.5% *vs*. 34.4%, p < 0.001), use of two or more drugs (66.2% *vs*. 43.0%, p < 0.001), and use of antibiotics (14.6% *vs*. 6.9%, p = 0.001), antifungals (9.4% *vs*. 4.0%, p = 0.013), antiparasitics (5.6% *vs*. 1.8%, p = 0.031) and antacids (8.6% *vs*. 3.6%, p = 0.024). Information about duration of treatment and medical advice was more complete with version A.

**Conclusion:**

The indication/drug-specific questions (questionnaire A) revealed a significantly higher prevalence of use of medicines – antibiotics, antifungals, antiparasitics and antacids – without compromising the completeness of the information.

## Background

Self-reported data are a common source of drug exposure information [1-4] and in many settings they provide the only method available for characterizing the use of medicines [5].

Modes of data collection by questionnaire may differ in several ways, including the methods for contacting respondents and for questionnaire delivery, and the administration of questions (*e.g*. the appearance of the questionnaire, the language used and the cultural adaptation and wording). These differences may affect the reliability and validity of the method [6-8].

Regarding drug utilization, questionnaire characteristics such as question wording and question or response order or format can be variously associated with exposure misclassification. Studies addressing the effect of questionnaire design on the recall of pharmacological treatments are heterogeneous in aim, method and quality, but are unanimous in concluding that indication- or drug-oriented questions reveal higher prevalences of drug utilization than open-ended questions [9]. However, obtaining data related to various drug categories frequently requires lengthy questionnaires including separate questions for each of the main pharmaceutical groups under study. It may not be possible or desirable to use large number of questions for this purpose, as that may substantially increase the size of the questionnaire and lead to poor acceptability and participant compliance. A short questionnaire may have the advantages of lower respondent burden and lower administrative costs, but its ability to characterize drug utilization fully is expected to be more limited. Shorter questionnaire versions can hardly include specific questions about the different pharmacological groups being assessed, which may compromise recall, and the potential for a lower burden may be attenuated by the use of more complex questions or a less attractive appearance, with many questions concentrated in a small area [10,11].

We hypothesized that a single-item questionnaire complemented with examples of the indications and medicines that are expected to be more frequent in the population studied could yield similar estimates of the prevalence of drug utilization to an extended multi-item questionnaire addressing the utilization of medicines for several indications. Therefore, our aim was to compare recall using two questionnaires differing in structure and length (long questionnaire including indication/drug-specific questions vs. short questionnaire including one open-ended question and several examples of indications and drugs).

## Methods

**Figure 1:**
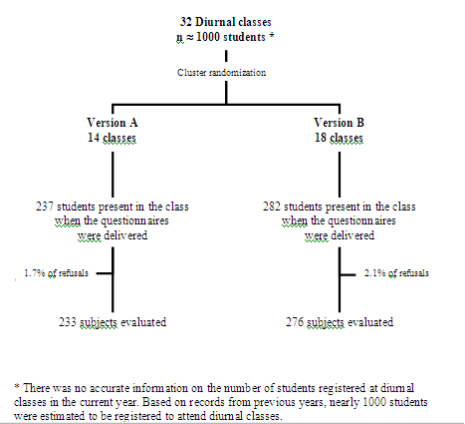
**Flowchart of the sampling strategies**.

Students enrolled at a private University in Maputo, Mozambique were investigated in April 2007. Socio-demographic details and information regarding the drugs used during the previous month (when applicable) were collected using a self-administered questionnaire presented to all diurnal classes, with the consent of the teachers. The questionnaire was given to all the students present in each class. No attempt was made to contact those who were absent. Ten students (1.9% of all invited) refused to participate (1.7% of those receiving questionnaire A and 2.1% of those receiving questionnaire B) and 509 were evaluated, approximately 50% of the total number of students in the institution who were enrolled in diurnal classes (Figure 1).

Two alternative versions (A and B) of the questionnaire, differing in question structure and length, were used to collect information. Both versions started with the same close-ended question "*Did you use any medication in the last month (including tablets, capsules, injections, ointments, ovules, syrups, etc.)?*" and gave similar general instructions to the participants. The questionnaires answered by the participants (versions A and B) were in Portuguese. English versions are provided as additional files 1 and 2.

Questionnaire A (*see *additional file 1: *questionnaire A*) was 4 pages long and included separate questions, with examples of specific drugs or medicines, for each of 12 indications/pharmacological groups, and one additional question for "other medicines" (see table 1). Subjects were asked to complete an open-ended table for each group, with the following information for each drug/medicine: brand or generic name, duration of treatment, medical advice and intended purpose. The indications/pharmacological groups for which specific questions were asked in questionnaire A and the examples given to the participants were selected from the medicines most frequently consumed by this population, as observed in a previous survey in the same setting [12]. The order of the questions in questionnaire A took into account the frequency of consumption (the most frequently-consumed medicines were the first to be asked about) and the similarity of indications/pharmacological groups (*e.g*. although antimalarial drugs were rarely consumed during the previous month, the question about them was placed near questions about the use of other anti-infectious drugs).

**Table 1:** Indication/Pharmacological groups and examples presented in both questionnaire versions, and subsequent classification according ATC nomenclature.

Indication/Pharmacological group	Examples	Classification (level II ATC subgroups)
Medications for treatment of pain and inflammation	Paracetamol, Voltaren^®^/diclofenac, ibuprofen, etc.	Analgesics (M01: Anti-inflammatory and anti-rheumatic drugs, non steroids, N02: Analgesics, B01: Antithrombotic agents)

Medications for treatment of flu or cold	Cêgripe^®^, Constipal^®^, Corenza^® ^C	Analgesics

Antibiotics	amoxicillin, tetracycline, co-trimoxazol, metronidazol, etc.	Antibiotics (J01: Antibacterials for systemic use)

Antimicotics/antifungals for treatment of infections	Canesten^®^, Clotrimazol^®^, Quadriderme^®^, Nalbix^®^, etc	Antifungals (ATC D01: Antifungals for dermatological use; G01: Gynecological antiinfectives and antiseptics; D07: Corticosteroids, dermatological preparations, combinations with antibiotics, J02: Antimicotics for systemic use)

Antimalarials	artemisine + fansidar^®^, etc	Antimalarials (ATC P01B: Antimalarials)

Antiparasitics	albendazol, mebendazol, etc	Antiparasitics (P01, except P01B: Antiprotozoals; P02: Antihelmintics)

Vitamins and minerals	multivitamins, complex B, ferrous salt, vitamin C, etc.	Vitamins (ATC A11: Vitamins, B03: Antianemic preparations)

Antiasthmatics	salbutamol/Ventilan^®^, aminofiline, beclomethasone, prednisolone, etc.	Antiasthmatics (R03: Drugs for obstructive airway diseases, H02: CCT for systemic use

Antihistamines	clorfeniramine, loratadine, Claritine^®^, etc.),	Anti-histamines (ATC R06: Antihistamines for systemic use)

Oral contraceptives/«pill»	Diane^® ^35, Microginon^®^, etc.	Hormonal contraceptives for systemic use (ATC G03: Sex hormones and modulators of the genital system; L02: Endocrine Therapy)

Antitussives and/or expectorants	Benilyn^®^, Diacol^®^, Benetussin^®^, Tosseque^®^, sodium benzoate, etc.	Antitussives (ATC R05: Cough and cold preparations)

Medications for gastric problems	omeprazole, cimetidine, ranitidine, ENO^®^- fruit salts, aluminium hydroxide, Rennie^®^, Kompensan^®^, etc.	Antacids (ATC A02: Drugs for acid related disorders)

Others	including all the drugs not included in the previously-described groups	

Questionnaire B (*see *additional file 2: *questionnaire B*) was one page long and included a single open-ended question to cover overall drug consumption, preceded by a short passage of text including the same examples provided in each of the questions from questionnaire A. Subjects were asked to complete a table similar to the ones described for questionnaire A.

Each medicine mentioned in questionnaires A or B was coded to the second level of classification (therapeutic subgroup) of the WHO Anatomical Therapeutic Chemical classification (ATC) [13]. The pharmacological groups/indications for which there were specific questions in questionnaire A and the corresponding ATC subgroups are shown in table 1. For data analysis, the medicines classified as "medications for treatment of pain and inflammation" or as "medications for treatment of flu or cold" were considered jointly as "analgesics".

Information about medical advice (to assess the use of medication following recent or past medical advice) and duration of treatment was analysed separately for drugs from each pharmacological group. For subjects using more than one medicine in the same group, the mean duration of treatment was used for analysis. For each pharmacological group, the results regarding medical advice are presented as the percentage of subjects who had used at least one medicine under a physician's advice.

To test the hypothesis that the two differently-structured questionnaires yielded the same prevalence of drug utilization, one version was randomly assigned to each of the 32 diurnal classes using a list of random numbers generated with EPi Info software, version 6.04d [14]. Questionnaires A and B were designated to 14 and 18 classes, respectively. All subjects within the same classroom received the same questionnaire version (A: 233 subjects; B: 276 subjects).

The choice of a one-month recall period was based on the expected frequency of drug utilization in this population, which was informed by a previous survey in the same setting [12]. This sample allows differences in the estimates of the two questionnaires to be detected with a power of 80%, for a 95% confidence level, when the magnitude of the difference corresponds to a risk ratio of two, and the proportion of drug use is above 10%, considering a design effect as high as 1.15 (which would be obtained for an average cluster size of 15 and an intra-cluster correlation coefficient of 0.01) [15]. The assumptions about the expected design effect and average cluster size are supported by a previous similar study in the same setting [16].

Logistic regression with robust standard errors (allowing for clustering by classroom) was used to compare the two groups (questionnaire A and questionnaire B) in terms of socio-demographic characteristics and medication used during the previous month [17]. All the tables present p-values corresponding to comparisons with no adjustment of covariates. The groups of medicines are presented in the tables in the order in which they appeared in Questionnaire A. Data were analyzed using STATA^®^, version 9.2.

The National Ethics Committee of Mozambique approved the survey protocol. Students were asked to read an informed consent form stating the general objectives of the study and data collection methods, in agreement with the Declaration of Helsinki. Only students who signed the informed consent form were allowed to participate and were asked to complete the questionnaire.

## Results

There were no statistically significant differences in the characteristics of the participants answering questionnaires A or B in terms of socio-demographic characteristics or the percentage of subjects who had used at least one medicine during the previous month (67.4% in both questionnaire versions) (Table 2).

**Table 2:** Characteristics of participants evaluated using questionnaires A (14 classrooms, 233 subjects) and B (18 classrooms, 276 subjects).

		Questionnaire		
			
	Number of subjects*	A n (%)	B n (%)	P
Age (% above 20)	489	101 (44.9)	103 (39.0)	0.557
Sex (% male)	497	80 (35.2)	104 (38.5)	0.728
Study area (% health related)	509	77 (33.0)	88 (31.9)	0.949
Grade (% above 2^nd ^grade)	509	133 (57.1)	111 (40.2)	0.404
Users of at least one medicine during the previous month	509	157 (67.4)	186 (67.4)	0.999

* The number of subjects may differ between the variables owing to missing data.

The median number of different pharmacological groups used by each subject (2 *vs*. 1), and the percentage of participants reporting the use of drugs from two or more pharmacological groups, were higher among those completing questionnaire A (60.5% *vs*. 34.4%, p < 0.001). Also, questionnaire A revealed a greater median number of medicines used per person among drug users (2 *vs*. 1) and a greater percentage of participants reporting the use of two or more drugs (66.2% *vs*. 43.0%, p < 0.001). However, the median number of drugs reported for all groups was 1 in both questionnaires, except for antimalarials (A *vs*. B: 2 *vs*.1).

Participants completing questionnaire A recalled use of the following drugs more frequently: antibiotics (14.6% *vs*. 6.9%, p = 0.001), antifungals (9.4% *vs*. 4.0%, p = 0.013), antiparasitics (5.6% vs. 1.8%, p = 0.031) and antacids (8.6% vs. 3.6%, p = 0.024). However, no statistically significant differences were observed for the remaining drug groups (Table 3).

**Table 3:** Medication used during the previous month, according to the type of questionnaire (n = 509).

	Questionnaire		
	A n (%)	B n (%)	P
Analgesics	114 (48.9)	127 (46.0)	0.634
Antibiotics	34 (14.6)	19 (6.9)	0.001
Antifungals	22 (9.4)	11 (4.0)	0.013
Antiparasitics	13 (5.6)	5 (1.8)	0.031
Antimalarials	3 (1.3)	5 (1.8)	0.690
Vitamins	38 (16.3)	28 (10.1)	0.118
Antiasthmatics	7 (3.0)	10 (3.6)	0.666
Antihistamines	34 (14.6)	34 (12.3)	0.362
Contraceptives *	14 (9.5)	14 (8.4)	0.742
Antitussives	6 (2.6)	2 (0.7)	0.118
Antacids	20 (8.6)	10 (3.6)	0.024
Other drugs	22 (8.0)	18 (7.7)	0.922

* Information on the use of contraceptives refers only to the female participants.

When the data were analysed separately for males and females, the relationship between the prevalences of drug use were essentially the same for both sexes (Table 4).

**Table 4:** Medication used by women and men during the previous month, according to the type of questionnaire.

	Women (n = 313)			Men (n = 184)		
				
	Questionnaire			Questionnaire		
				
	A n (%)	B n (%)	P	A n (%)	B n (%)	P
Analgesics	75 (51.0)	88 (53.0)	0.795	37 (46.2)	38 (36.5)	0.109
Antibiotics	21 (14.3)	12 (7.2)	0.007	11 (13.8)	7 (6.7)	0.186
Antifungals	14 (9.5)	7 (4.2)	0.013	7 (8.8)	4 (3.8)	0.115
Antiparasitics	12 (8.2)	3 (1.8)	0.002	1 (1.2)	2 (1.9)	0.685
Antimalarials	2 (1.4)	5 (3.0)	0.445	1 (1.2)	0 (0.0)	---
Vitamins	28 (19.0)	22 (13.2)	0.282	9 (11.2)	5 (4.8)	0.111
Antiasthmatics	6 (4.1)	6 (3.6)	0.812	1 (1.2)	4 (3.8)	0.325
Antihistamines	23 (15.6)	22 (13.2)	0.541	10 (12.5)	12 (11.5)	0.802
Antitussives	3 (2.0)	1 (0.6)	0.268	3 (3.8)	1 (1.0)	0.220
Antacids	17 (11.6)	8 (4.8)	0.048	3 (3.8)	2 (1.9)	0.322
Other drugs	13 (8.8)	10 (6.0)	0.316	3 (3.8)	4 (3.8)	0.977

The proportion of subjects who did not provide information about the duration of previously reported treatments differed across pharmacological groups, but tended to be much higher among participants completing questionnaire B. The duration of treatment reported by the participants ranged from 1 day for antimalarials and 2 days for analgesics to 15 days for antiasthmatics and 1 month for oral contraceptives (Table 5).

**Table 5:** Information about the duration of treatment with medication used during the previous month, according to the type of questionnaire.

	Missing information regarding duration of treatment, n (%)		Median duration of treatment (days)	
	Questionnaire		Questionnaire	
	A	B	A	B
Analgesics	9 (8.0)	21 (17.1)	3	2
Antibiotics	2 (5.9)	4 (21.0)	7	7
Antifungals	1 (4.5)	4 (36.4)	14	7
Antiparasitics	2 (15.4)	0 (0.0)	3	3
Antimalarials	0 (0.0)	2 (40.0)	3.5	1
Vitamins	3 (7.9)	7 (25.0)	15	7
Antiasthmatics	0 (0.0)	3 (30.0)	3.5	15
Antihistamines	4 (11.8)	13 (38.2)	4	3
Contraceptives *	2 (14.3)	0 (0.0)	30 †	30 †
Antitussives	0 (0.0)	0 (0.0)	5	10.5
Antacids	1 (5.0)	2 (20.0)	3	4.5

* Information on the use of contraceptives refers only to the female participants; † questionnaire completion examples included oral contraceptives with an exposure period defined as daily, which probably induced respondents to frame a 30-day duration of treatment.

The proportion of subjects who did not provide information about treatment under medical advice during the preceding month differed across pharmacological groups, but tended to be much higher among participants completing questionnaire B. There were also clear differences in the percentage of subjects who received treatment under medical advice, but no systematic differences were observed according to the questionnaire used (Table 6).

**Table 6:** Information about the medicines used under medical advice during the previous month, according to the type of questionnaire.

	Missing information regarding medical advice, n (%)		Subjects who had used at least one medicine under medical advice, n (%)	
	Questionnaire		Questionnaire	
	A	B	A	B
Analgesics	5 (4.4)	15 (11.8)	34 (31.2)	22 (19.6)
Antibiotics	2 (5.9)	3 (15.8)	22 (68.8)	13 (81.3)
Antifungals	0 (0.0)	3 (27.3)	16 (72.7)	5 (62.5)
Antiparasitics	0 (0.0)	0 (0.0)	7 (53.9)	3 (60.0)
Antimalarials	0 (0.0)	2 (40.0)	3 (100.0)	1 (33.3)
Vitamins	2 (5.3)	8 (28.6)	19 (52.8)	13 (65.0)
Antiasthmatics	1 (14.3)	4 (40.0)	5 (83.3)	6 (100.0)
Antihistamines	1 (2.9)	8 (23.5)	7 (21.2)	7 (26.9)
Contraceptives *	1 (7.1)	3 (21.4)	11 (84.6)	8 (72.7)
Antitussives	1 (16.7)	0 (0.0)	2 (40.0)	2 (100.0)
Antacids	1 (5.0)	1 (10.0)	5 (26.3)	9 (32.1)

* Information on the use of contraceptives refers only to the female participants.

## Discussion

The longer questionnaire version, which included indication/drug-specific questions, revealed a significantly higher prevalence of use of antibiotics, antifungals/antimicotics, antiparasitics and antacids. For the remaining drug groups, recall did not differ significantly between questionnaire versions. Participants receiving the questionnaire version relying on a single open-ended question tended to provide less complete information about treatment duration and medical advice.

The differences in the estimates obtained with the two questionnaire versions were not the same across all pharmacological groups or drugs. This is consistent with previous finding in studies addressing the effect of questionnaire design on the recall of pharmacological treatments [16,18,19].

Ademi et al. [20] tested different question formats (frequency measure; symptom-oriented question; open-ended question), in a substantially different population, to quantify the prevalence of use of analgesics during the preceding seven days. The higher prevalence was obtained by asking the respondents to name the medicines they had used during the previous week, but the open-ended item was the last of three questions asked sequentially.

For antibiotics and antifungals/antimicotics the estimated prevalence differed significantly between the two questionnaire structures. This was also noted by Neutel et al. [19] who observed that antibiotics were better recalled when asked about by specific name rather than by drug category. Our study revealed no differences between the two questionnaire versions in assessing antimalarials, which may be because malaria is a very frequent severe disease in this setting, so we would not expect the participants to forget malaria treatments used within one month of the interview.

For pharmacological groups such as the antiasthmatics, no differences between questionnaire versions were observed in the prevalence of use, perhaps because these drugs are used to treat a chronic condition, unlikely to be forgotten by the participants, and recalled regardless of the questionnaire format used. For vitamins or antitussives, the differences observed were not significant but were consistent in men and women, suggesting that the recall may again be affected by the differences in questionnaire design.

The population evaluated in our study was relatively homogeneous in age and education and does not adequately represent Mozambican university students, still less the general population. Studies assessing the influence of education on recall in drug utilization have concluded that agreement between questionnaires and information retrieved from record data increases with education [21,22], although less educated subjects tend to use medicines more than better educated ones [1]. We may expect even greater differences between the results obtained with versions A and B of the questionnaire among older or less well-educated participants. The results from surveys based on self-reporting of drug intake among such populations need an even more cautious interpretation.

The internal validity of the present study is not compromised by our option for a one-month recall period, since it applied to both the questionnaire versions compared. Choosing a shorter recall period (*e.g*. two days or a week) would have contributed to more accurate recall of the medicines to which the participants had been exposed, but also to a lower prevalence of use. Conversely, a longer recall period (*e.g*. six months or one year) would have yielded worse recall [23] but a higher proportion of subjects using medicines. Regarding external validity, we may expect a structured questionnaire with specific questions about indication/pharmacological group to have more impact on the recall of medicines used for acute conditions, but a negligible impact when recall is only required over shorter periods.

Both groups (A and B) answered the questionnaire at the same time, so seasonal fluctuations on the intake of medicines for flu symptoms or malaria cannot be expected to have compromised internal validity. It may not be possible to generalize our results to studies conducted at other times of the year since the differences between questionnaire's versions may be related to the frequency of consumption of medicines used for seasonally variable conditions. We think it is unlikely that the recall of malaria treatment would be substantially influenced by questionnaire design, given the severity of the disease. For the remaining pharmacological groups used for acute conditions, our results do not suggest differences dependent on the frequency of consumption, beyond random variation. Also, the enhanced recall of drug utilization obtained using indication- and drug-oriented questions seems to be independent of the frequency of drug use [18].

The specificity of the questions about pharmacological treatments is known to contribute to more accurate estimates of medicine use [9]. However, since it is not feasible to write all possible examples of medicines for each indication/pharmacological group, recall may differ between the examples provided and medicines that are not listed. We tried to overcome this limitation by asking simultaneously about the use of medicines for specific indications/pharmacological groups and providing examples of the medicines most commonly used in this population. Moreover, the same sets of examples were used in both versions to ensure comparability in this aspect of questionnaire design.

Since the same medicine can be used for different purposes, respondents may become confused about how to respond when indications and example medicines are provided side by side. This is particularly important for drugs such as paracetamol, which may be used both for "pain and inflammation" and for symptomatic "treatment of flu or cold". We opted to use two separate questions for these indications, expecting to improve recall, and asked participants for the intended utilization of each medicine, allowing them to be classified correctly in this regard. In addition, although we had two separate questions for medicines used for "pain and inflammation" and "flu or cold", we opted to analyze these groups together, overcoming the potential misclassification.

No records were available to check the reported drug utilization against the prescribed and non-prescribed drug data, so no inferences could be drawn about the absolute validity of exposure recall using questionnaires A or B. However, studies comparing questionnaires with pharmacy data as a gold standard have shown that drug-oriented questionnaires yield better agreement than data collected by open-ended questions [24,21], and the design used for questionnaire A is expected to provide more accurate estimates of drug utilization [18,21]. Also, several studies have indicated that in drug utilization studies the prevalence estimates obtained from questionnaire surveys are more reliable than information retrieved from medical or pharmacy records, especially for assessing over-the-counter drugs and medication misuse [1,22,25-27]. Self-medication reports may be a useful instrument, together with medical prescriptions and pharmacy records, for measuring the efficacy of the drugs used [28].

A concern when using a long questionnaire such as version A is the burden that it imposes to the participant, requiring a long time to answer the questions, which may result in a larger proportion of refusals and in missing data among respondents [7]. On the other hand, the physical layout of a questionnaire is important for increasing response rates as it is sometimes easier to complete a questionnaire that, despite being longer, is clearer in structure [29]. In our study, the time taken by the participants to complete each questionnaire version could not be quantified, but the longer version is not necessarily expected to take more time to complete as it does not differ from the shorter one in the level of detail required. Also, the indication-oriented questions in version A may have been perceived as easier to answer because they were more structured, facilitating recall of previous treatments. The proportion of refusals was low and similar for both questionnaire versions (1.7% for version A and 2.1% for version B), probably because the questionnaires were completed in the classroom. Therefore, in the present study, missing information may be a better indicator of the acceptability of the questionnaire than the proportion of refusals.

The proportion of subjects with missing information about the duration of treatment or medical advice was even lower among those receiving the 4-page questionnaire, suggesting that both versions were well accepted by the participants and that the layout of the longer questionnaire facilitated more complete recall.

We found that a substantial proportion of students used medicines without medical advice, which would not be identified in a study based on doctor-generated or pharmacy-based prescription records. This finding has implications for studies relying on such databases and raises the concern that such information includes false positive «exposures» (prescriptions issued or completed but not consumed).

In self-administered questionnaires, the first options in a list of answering options are more likely to be chosen (response choice order effects [6]). We could eventually expect a similar phenomenon in the examples provided in questionnaire B, or question order effects in questionnaire A. The former would lead to larger differences between questionnaire versions, the latter to smaller differences. However, no consistent pattern was observed across the last groups provided as examples (version B) or specifically asked (version A), and the different characteristics of the last indications/pharmacological groups preclude such an interpretation of the findings.

## Conclusion

Our results add to previous research by demonstrating that questionnaire structure and layout contribute to differences in recall of pharmacological treatments, even when the content of the questionnaire and the type of questions are virtually the same. We also demonstrated that a larger questionnaire does not necessarily lead to less complete recall.

Future research on this topic should assess the impact of data collection instruments across populations with different patterns of medicine utilization and ability to recall usage over different periods.

For a proper interpretation of data from drug utilization studies it is essential to take into account the characteristics of the instruments used for data collection, and their potential impact on the validity of the estimates for different pharmacological groups and across settings.

## Competing interests

The authors declare that they have no competing interests.

## Authors' contributions

HG contributed to the analysis and interpretation of data and wrote the first version of the manuscript. SC contributed to the data analysis and revision of the manuscript. NL contributed to the conception and design of the study and critically revised the manuscript for intellectual content. All the authors read and approved the final version of the manuscript for publication.

## Pre-publication history

The pre-publication history for this paper can be accessed here:

http://www.biomedcentral.com/1471-2288/9/45/prepub

## Supplementary Material

Additional file 1 **Questionnaire A**. The data represent the version A of the questionnaire used in this study. Click here for file

Additional file 2 **Questionnaire B**. The data represent the version B of the questionnaire used in this study. Click here for file
