# Supplementary material for: Effect of questionnaire structure on recall of drug utilization in a population of university students
Source: BMC Med Res Methodol. 2009 Jun 29;9:45. doi: 10.1186/1471-2288-9-45 (PMC2713272; doi:10.1186/1471-2288-9-45)
Supplement: Additional file 1 — Questionnaire A. The data represent the version A of the questionnaire used in this study. [file 1471-2288-9-45-S1.doc]

# 1. Did you use any medication in the last month (including tablets, capsules, injections, ointments, ovules, syrups, etc.)?

# 0 ⬜ no _1_ ⬜ yes

# *If you answered no, go to question 15 (at the end of the questionnaire).*

*If you answered yes, please go to the next question.*

Please fill in the next tables according to this example

| Name of the medicine or drug | Duration of treatment (in days) | Medical advice  (yes or no) | Reason for using |
| --- | --- | --- | --- |
| vvvvvvvvDiane35vvvvvvvvv | Everyday | Yes | Prevent pregnancy |
| Vitamins and minerals | ± 15 days | No | Fatigue due to exams |
| Salbutamol | SOS/urgency | Yes | Asthma |
| Canesten (cream) | 15 days | No | Infection |
| Artemisinine+fansidar | Taken once | No | Malaria |

If you don’t remember the medication, please describe the type of medication and what it is/was used for.

If you only know to answer to part of the questions that we are asking you, please answer to those that you know how to answer (please leave what you can’t or don’t remember in blank).

# 2. In the last month did you use some medication (including tablets, capsules, injections, ointments, ovules, syrups) for treatment of pain or inflammation (e.g.: voltaren/diclofenac, paracetamol, ibuprofen, etc.)?

# 0 ⬜ no _1_ ⬜ yes

# *If you answered no, go to question 3.*

*If you answered yes, please* fill in the next table with the medication(s) that you used.

| Name of the medicine or drug | Duration of treatment (in days) | Medical advice  (yes or no) | Reason for using |
| --- | --- | --- | --- |
| vvvvvvvvvvvvvvvvvvvvvvvvvv | Vvvvvvvvvv | Vvvvvvvvv | vvvvvvvvvvvvvvvvvvvvvvvvvvvvvvvvv |
|  |  |  |  |

# 3. In the last month did you use some medication for treatment of flu or cold (e.g.: Cêgripe, Constipal, Corenza C, etc.)?

# 0 ⬜ no _1_ ⬜ yes

# *If you answered no, go to question 4.*

*If you answered yes, please* fill in the next table with the medication(s) that you used.

| Name of the medicine or drug | Duration of treatment (in days) | Medical advice  (yes or no) | Reason for using |
| --- | --- | --- | --- |
| vvvvvvvvvvvvvvvvvvvvvvvvvv | Vvvvvvvvvv | Vvvvvvvvv | vvvvvvvvvvvvvvvvvvvvvvvvvvvvvvvvv |
|  |  |  |  |

# 4. In the last month did you use some antibiotic (including tablets, capsules, injections, ointments) for treatment of infections (e.g. amoxicillin, tetracycline, co-trimoxazol, metronidazol, etc), not including antimalarials?

# 0 ⬜ no _1_ ⬜ yes

# *If you answered no, go to question 5.*

*If you answered yes, please* fill in the next table with the medication(s) that you used.

| Name of the medicine or drug | Duration of treatment (in days) | Medical advice  (yes or no) | Reason for using |
| --- | --- | --- | --- |
| vvvvvvvvvvvvvvvvvvvvvvvvvv | Vvvvvvvvvv | Vvvvvvvvv | vvvvvvvvvvvvvvvvvvvvvvvvvvvvvvvvv |
|  |  |  |  |

# 5. In the last month did you use some antifungal (ovules, ointments, tablets, capsules, injections), for treatment of infections (e.g. Canesten, Clotrimazol, quadriderme, Nalbix, etc.)?

# 0 ⬜ no _1_ ⬜ yes

# *If you answered no, go to question 6.*

*If you answered yes, please* fill in the next table with the medication(s) that you used.

| Name of the medicine or drug | Duration of treatment (in days) | Medical advice  (yes or no) | Reason for using |
| --- | --- | --- | --- |
| vvvvvvvvvvvvvvvvvvvvvvvvvv | Vvvvvvvvvv | Vvvvvvvvv | vvvvvvvvvvvvvvvvvvvvvvvvvvvvvvvvv |
|  |  |  |  |

# 6. In the last month did you use some antimalarial (e.g. artemisinine+fansidar, etc.)?

# 0 ⬜ no _1_ ⬜ yes

# *If you answered no, go to question 7.*

*If you answered yes, please* fill in the next table with the medication(s) that you used.

| Name of the medicine or drug | Duration of treatment (in days) | Medical advice  (yes or no) | Reason for using |
| --- | --- | --- | --- |
| vvvvvvvvvvvvvvvvvvvvvvvvvv | Vvvvvvvvvv | Vvvvvvvvv | vvvvvvvvvvvvvvvvvvvvvvvvvvvvvvvvv |
|  |  |  |  |

7. In the last month did you use antiparasitics (e.g. albendazol, mebendazol, etc.)?

# 0 ⬜ no _1_ ⬜ yes

# *If you answered no, go to question 8.*

*If you answered yes, please* fill in the next table with the medication(s) that you used.

| Name of the medicine or drug | Duration of treatment (in days) | Medical advice  (yes or no) | Reason for using |
| --- | --- | --- | --- |
| vvvvvvvvvvvvvvvvvvvvvvvvvv | Vvvvvvvvvv | Vvvvvvvvv | vvvvvvvvvvvvvvvvvvvvvvvvvvvvvvvvv |
|  |  |  |  |

8. In the last month did you use vitamins and minerals (e.g. multivitamins, complex B, ferrous salt, vitamin C etc.)?

# 0 ⬜ no _1_ ⬜ yes

# *If you answered no, go to question 9.*

*If you answered yes, please* fill in the next table with the medication(s) that you used.

| Name of the medicine or drug | Duration of treatment (in days) | Medical advice  (yes or no) | Reason for using |
| --- | --- | --- | --- |
| vvvvvvvvvvvvvvvvvvvvvvvvvv | Vvvvvvvvvv | Vvvvvvvvv | vvvvvvvvvvvvvvvvvvvvvvvvvvvvvvvvv |
|  |  |  |  |

9. In the last month did you use some antiasthmatic (e.g. salbutamol/Ventilan, aminofiline, becometazol, prednisolone, etc)?

# 0 ⬜ no _1_ ⬜ yes

# *If you answered no, go to question 10.*

*If you answered yes, please* fill in the next table with the medication(s) that you used.

| Name of the medicine or drug | Duration of treatment (in days) | Medical advice  (yes or no) | Reason for using |
| --- | --- | --- | --- |
| vvvvvvvvvvvvvvvvvvvvvvvvvv | Vvvvvvvvvv | Vvvvvvvvv | vvvvvvvvvvvvvvvvvvvvvvvvvvvvvvvvv |
|  |  |  |  |

10. In the last month did you use some antihistamine (ex. Clorfeniramine, loratidine, claritine, etc.)?

# 0 ⬜ no _1_ ⬜ yes

# *If you answered no, go to question 11.*

*If you answered yes, please* fill in the next table with the medication(s) that you used.

| Name of the medicine or drug | Duration of treatment (in days) | Medical advice  (yes or no) | Reason for using |
| --- | --- | --- | --- |
| vvvvvvvvvvvvvvvvvvvvvvvvvv | Vvvvvvvvvv | Vvvvvvvvv | vvvvvvvvvvvvvvvvvvvvvvvvvvvvvvvvv |
|  |  |  |  |

11. In the last month did you use some oral contraceptives/«pill» (ex. Diane 35, Microginon, etc?

# 0 ⬜ no _1_ ⬜ yes

# *If you answered no, go to question 12.*

*If you answered yes, please* fill in the next table with the medication(s) that you used.

| Name of the medicine or drug | Duration of treatment (in days) | Medical advice  (yes or no) | Reason for using |
| --- | --- | --- | --- |
| vvvvvvvvvvvvvvvvvvvvvvvvvv | Vvvvvvvvvv | Vvvvvvvvv | vvvvvvvvvvvvvvvvvvvvvvvvvvvvvvvvv |
|  |  |  |  |

12. In the last month did you use antitussives and/or expectorants (ex. Benilyn, Diacol, Benetussin, Tosseque, Sodium benzoate, etc.)?

# 0 ⬜ no _1_ ⬜ yes

# *If you answered no, go to question 13.*

*If you answered yes, please* fill in the next table with the medication(s) that you used.

| Name of the medicine or drug | Duration of treatment (in days) | Medical advice  (yes or no) | Reason for using |
| --- | --- | --- | --- |
| vvvvvvvvvvvvvvvvvvvvvvvvvv | Vvvvvvvvvv | Vvvvvvvvv | vvvvvvvvvvvvvvvvvvvvvvvvvvvvvvvvv |
|  |  |  |  |

13. In the last month did you use some medication for gastric problems (ex. omeprazole, cimetidine, ranitidine, ENO – fruits salts, aluminium hydroxide, Rennie, Kompensan etc.)?

# 0 ⬜ no _1_ ⬜ yes

# *If you answered no, go to question 14.*

*If you answered yes, please* fill in the next table with the medication(s) that you used.

| Name of the medicine or drug | Duration of treatment (in days) | Medical advice  (yes or no) | Reason for using |
| --- | --- | --- | --- |
| vvvvvvvvvvvvvvvvvvvvvvvvvv | Vvvvvvvvvv | Vvvvvvvvv | vvvvvvvvvvvvvvvvvvvvvvvvvvvvvvvvv |
|  |  |  |  |

14. In the last month did you use some medication (including tablets, capsules, injections, ointments, ovules, syrups, etc.), other than those that you reported in the previous questions?

# 0 ⬜ no _1_ ⬜ yes

# *If you answered no, go to question 15.*

*If you answered yes, please* fill in the next table with the medication(s) that you used.

| Name of the medicine or drug | Duration of treatment (in days) | Medical advice  (yes or no) | Reason for using |
| --- | --- | --- | --- |
| vvvvvvvvvvvvvvvvvvvvvvvvvv | Vvvvvvvvvv | Vvvvvvvvv | vvvvvvvvvvvvvvvvvvvvvvvvvvvvvvvvv |
|  |  |  |  |

Socio-demgraphics

15. What is your sex? _0_ ⬜ female _1_ ⬜ male

16. What is your age? |___|___| years

17. What is your ethnicity? _1_ ⬜ black _2_ ⬜ white _3_ ⬜ mixed _4_ ⬜ indian _5_ ⬜ other

Thank you for your cooperation!
