# Supplementary material for: Effect of questionnaire structure on recall of drug utilization in a population of university students
Source: BMC Med Res Methodol. 2009 Jun 29;9:45. doi: 10.1186/1471-2288-9-45 (PMC2713272; doi:10.1186/1471-2288-9-45)
Supplement: Additional file 2 — Questionnaire B. The data represent the version B of the questionnaire used in this study. [file 1471-2288-9-45-S2.doc]

# 1. Did you use any medication in the last month (including tablets, capsules, injections, ointments, ovules, syrups, etc.)?

# 0 ⬜ no _1_ ⬜ yes

# *If you answered no, go to question 15 (at the end of the questionnaire).*

*If you answered yes, please go to the next question.*

Please fill in the next tables according to this example:

To better remind you, here are some examples of medications: Medicines for treatment of pain or inflammation (e.g.: voltaren/diclofenac, paracetamol, ibuprofen, etc.); medication for treatment of flu or cold (e.g.: Cêgripe, Constipal, Corenza C, etc.); antibiotics (e.g. amoxicillin, tetracycline, co-trimoxazol, metronidazol, etc), antifungals fo treatment of infections (e.g. Canesten, clotrimazol, Quadriderme, Nalbix, etc.); antimalarials (e.g. artemisinine+fansidar, etc.); anti-parasitics (e.g. albendazol, mebendazol, etc.); vitamins and minerals (e.g. multivitamins, complex B, ferrous salt, vitamin C, etc.); anti-asthmtics (e.g. salbutamol/Ventilan, aminofiline, becometazol, prednisolone, etc); anti-histaminics (e.g. clorfeniramine, loratidine, claritine, etc.); oral contracceptives/”pill”(e.g. Diane 35, Microginon, etc.); antitussives and/or expectorants (e.g. Benilyn, Diacol, Benetussin, Tosseque, Sodium benzoate, etc.); or medicines for gastric problems (e.g. omeprazol, cimetidine, ranitidine, ENO-fruit salt, aluminium hydroxide, Rennie, Kompensan, etc.)

If you don’t remember the medication, please describe the type of medication and what it is/was used for.

If you only know to answer to part of the questions that we are asking you, please answer to those that you know how to answer (please leave what you can’t or don’t remember in blank).

|  | Name of the medicine or drug | Duration of treatment (in days) | Medical advice  (yes or no) | Reason for using |
| --- | --- | --- | --- | --- |
| Example | vvvvvvvvDiane35vvvvvvvvv | Everyday | Yes | Prevent pregnancy |
| Example | Vitamins and minerals | ± 15 days | No | Fatigue due to exams |
| Example | Eritromicin | 8 days | Yes | Infection |
| Example | Canesten (cream) | 15 days | No | Infection |
| a) |  |  |  |  |
| b) |  |  |  |  |
| c) |  |  |  |  |
| d) |  |  |  |  |

Socio-demographics

15. What is your sex? _0_ ⬜ female _1_ ⬜ male

16. What is your age? |___|___| years

17. What is your ethnicity? _1_ ⬜ black _2_ ⬜ white _3_ ⬜ mixed _4_ ⬜ indian _5_ ⬜ other
